# Illinois State Veterinary Medical Association

**Published:** 1896-01

**Authors:** Albert Babb

**Affiliations:** Secretary


### ILLINOIS STATE VETERINARY MEDICAL ASSOCIATION.

THE thirteenth annual meeting was held in the Sherman House, Chicago, November 20 and 21, 1895. On roll-call a full attendance of members responded.

An impressive address was delivered by the President, Dr. M. Wilson, of Mendota, and other papers were presented as follows: "My First Cæsarean Section in the Cow," Dr. A. Babb; "Pneumonia," Dr. S. S. Baker; "Some Diseases of the Hock," Dr. W. J. Martin; "Soundness and Lameness," Dr. A. G. Alverson; "Bovine Tuberculosis," M. R. Trumbower; "Transmission of Disease," A. H. Baker. Many valuable points in therapeutics were brought out by the essayists and the excellent discussion.

The following were elected to membership: H. A. Pressler, Fairbury; A. J. Savage, Lichfield; E. S. Fry, Naperville; and Olof Schwarzkopf, Dean of the McKillip Veterinary College, Chicago.

The election of officers for the coming year resulted as follows: President, M. R. Trumbower, State Veterinarian, Sterling; Vice-President, J. L. Tyler, Chebanse; Secretary, Albert Babb (re-elected), Springfield; Treasurer, R. G. Walker, Chicago; Board of Censors: T. J. Gunning, Neponset; W. J. Martin, Kankakee; and Joseph Hughes, Chicago Veterinary College.

On motion, Galesburg was selected as the place for the next meeting, which will be held in about six months.

ALBERT BABB, A.B., M.D.C.,
Secretary.

